# Appropriate intradeltoid muscle needle penetration depth in vaccine administration: an MRI study in Thailand

**DOI:** 10.3389/fimmu.2023.1302891

**Published:** 2023-12-15

**Authors:** Teeranan Laohawiriyakamol, Pattira Boonsri, Khetnathee Phutphithak, Thamonchanok Niyomkarn, Prapakorn Klabklay, Chaiwat Chuaychoosakoon

**Affiliations:** Faculty of Medicine, Prince of Songkla University, Songkhla, Thailand

**Keywords:** deltoid, injection depth, subcutaneous, thickness, vaccination

## Abstract

**Objective:**

The objective of this study was to evaluate the appropriate vaccination needle penetration depth into the deltoid muscle to avoid injection-site complications from an inappropriate injection depth and/or injection site in the Thai population.

**Methods:**

This was a retrospective study using axial proton density-weighted images of MRI shoulders at the level of 2 fingerbreadths below the acromion process to measure the combined thickness of the skin, subcutaneous fat pad and deltoid muscle to evaluate the percentage of injections into the deltoid muscle with various needle penetration depths.

**Results:**

There were 509 MRI shoulder images of 222 males and 287 females (265 right shoulders and 244 left shoulders). The average body mass index and age were 24.54 ± 3.54 kg/m^2^ and 64.81 ± 10.20 years, respectively. Using a needle penetration depth of 12.7 mm (0.5 inches) achieved 100% of injections into the deltoid muscle.

**Conclusion:**

We recommend advancing the entire length of a 0.5-inch needle perpendicular to the skin at 2 fingerbreadths below the acromion process for adult intradeltoid vaccinations. This approach ensures optimal vaccine delivery and minimizes the risk of injection-related injuries.

## Introduction

**Figure 1:**
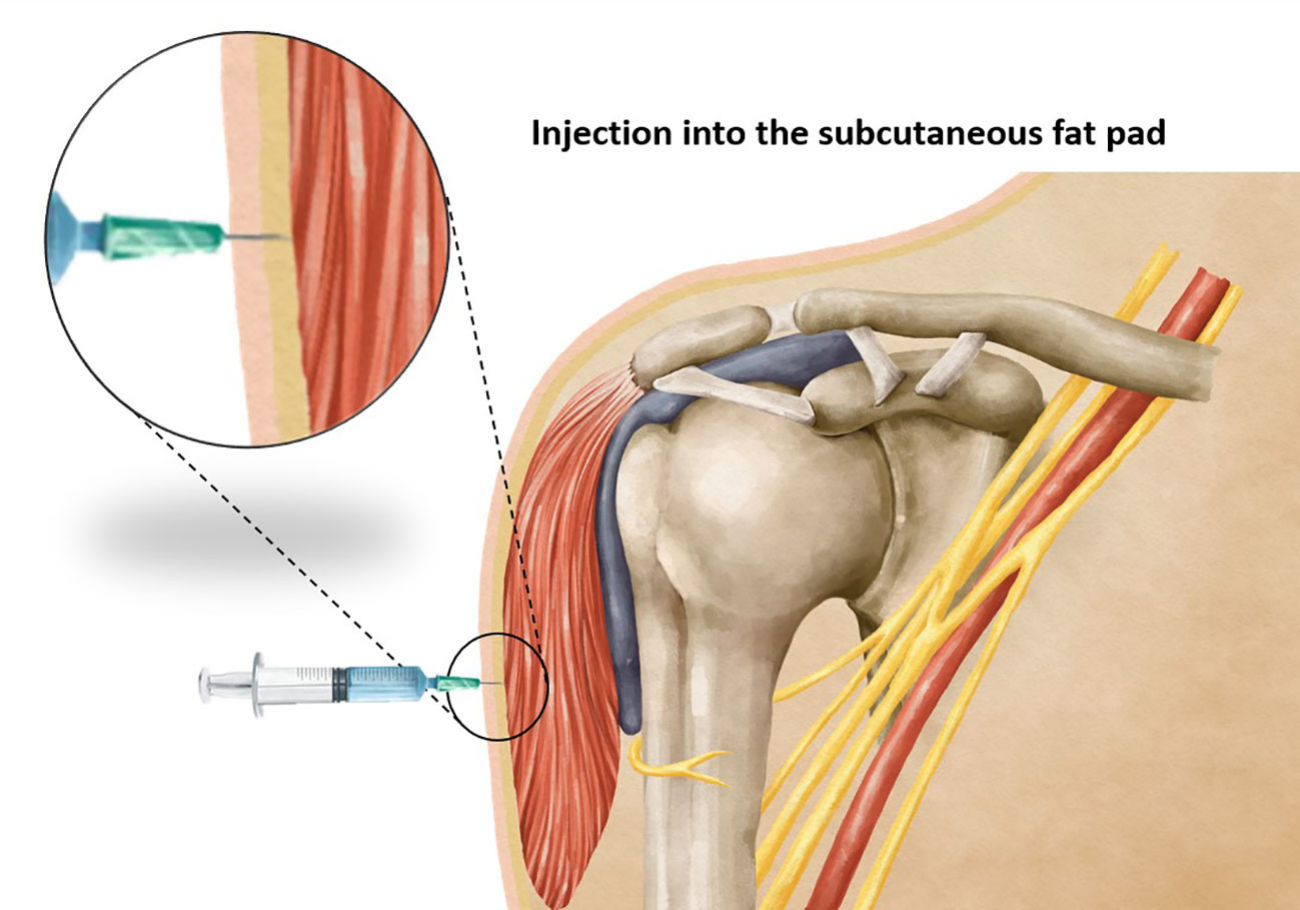
Showing an injection into the subcutaneous fat pad when using a too-short needle length.

**Figure 2:**
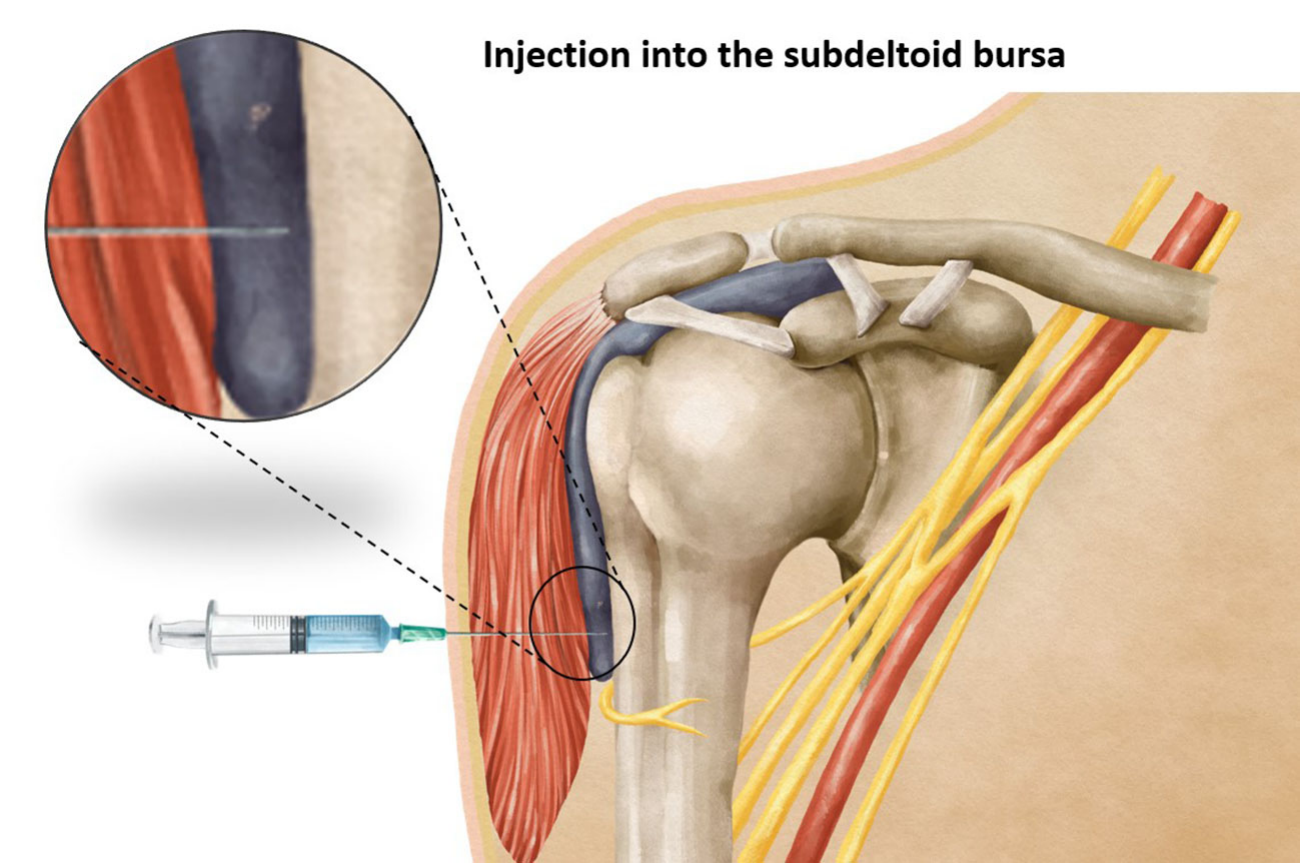
Showing an injection into the subdeltoid bursa when using a too-long needle length.

Vaccination stands as one of the most effective strategies in preventing the spread of infectious diseases. The precise administration of a vaccine requires careful attention to various factors, including the selection of an appropriate needle length to ensure accurate delivery of the vaccine at the intended site. Intramuscular vaccines, such as those for hepatitis A, hepatitis B, rabies, influenza, diphtheria toxoid, and coronavirus-19 (COVID-19) show optimal efficacy when administered intramuscularly (1, 2), predominantly within the deltoid muscle. It has been observed that using a needle that is too short may inadvertently lead to subcutaneous administration of the vaccine (**Figure 1**), resulting in reduced vaccine efficacy, seropositivity, and antibody titers compared to an intramuscular injection (3). On the other hand, the use of a too-long needle may lead to inadvertent injection into the subdeltoid bursa (**Figure 2**), potentially causing bursitis (4). Thus, careful attention to needle length selection during vaccine administration is important to ensure proper intramuscular deposition of the vaccine, thereby enhancing vaccine effectiveness and minimizing the risk of adverse events associated with subcutaneous or bursal injections.

As per the Centers for Disease Control and Prevention (CDC) guidelines, the correct injection site is 2 or 3 fingerbreadths below the acromion process. However, utilizing 2-3 fingerbreadths below the acromion process with an excessively long needle may increase the likelihood of injecting into the subdeltoid bursa, as this bursa can extend more than 5 cm below the acromion process. Notably, research findings indicate that using a distance of 3 fingerbreadths (57.69 mm) (5) below the acromion process may pose a risk of iatrogenic axillary nerve injury. Some studies have reported the presence of the axillary nerve at a location 5 cm below the acromion process (6–10). In summary, an injection site located 3 fingerbreadths below the mid-acromion process, when using a needle of excessive length, carries a risk of both axillary nerve injury and subdeltoid bursitis. Conversely, utilizing an injection site located 2 fingerbreadths below the mid-acromion process only poses a risk of subdeltoid bursitis, making this measurement more suitable for vaccination administration. Healthcare practitioners should exercise caution and employ appropriate needle lengths to minimize the risk of complications while adhering to the recommended injection site guidelines.

To date, there are no studies which have comprehensively evaluated the thickness of the subcutaneous fat pad and the thickness from the skin to the subdeltoid fascia, providing guidance for determining the optimal needle penetration depth for intradeltoid muscle vaccination at the landmark of 2 fingerbreadths below the mid-acromion process. Thus, the objective of this study was to measure the thickness of the subcutaneous fat pad and the thickness from the skin to the subdeltoid fascia, aiming to establish an ideal needle penetration depth that would avoid both subcutaneous and subdeltoid bursa delivery of the injectant at the landmark of 2 fingerbreadths below the acromion process, and secondly, to assess the risk of vaccine injection into the subcutaneous fat pad or subdeltoid bursa for the Thai population when following the CDC guidelines. The study hypothesized that a needle penetration depth of 0.5 inches (12.7 mm) would successfully reach the deltoid muscle while avoiding passage into the subdeltoid bursa, effectively traversing both the skin and subcutaneous fat pad. Additionally, it was anticipated that following the CDC guidelines for vaccination may result in the use of a needle length that is too long in our Thai patients, potentially leading to injection into the subdeltoid bursa.

## Methods

### Study design, population, and place

This retrospective study was approved by the Institutional Review Board of the Faculty of Medicine of Prince of Songkla University. The requirement for informed consent from the study subjects was waived by the Institutional Review Board due to the retrospective study design (REC 65-293-11-1). MRI images were examined and certain measurements made to assess the thickness of the skin, subcutaneous fat pad and deltoid muscle, in order to determine an appropriate needle penetration depth.

This study was an anatomical study based on patient MRIs, therefore there was no public involvement, and no direct patient involvement.

The study included 600 MRIs which had been taken between January 1, 2012, and July 31, 2022, of which the MRIs of 91 patients who had had bone or soft tissue tumor, a fracture around the shoulder joint or a history of shoulder surgery, or were aged less than 18 years were excluded. All of the MRI images were anonymized, therefore the patients could not be contacted before or after the study.

To conduct the analysis, the MRI shoulder images were carefully selected and measured using a specialized Picture Archiving and Communication System (PACS) workstation. In our study, we simulated the vaccination conditions by selecting the injection site as 2 fingerbreadths below the mid-acromial process and administering the simulated injection perpendicular to the skin. This approach aimed to replicate the standard vaccination procedure for accurate and consistent results in our simulation.

**Figure 3:**
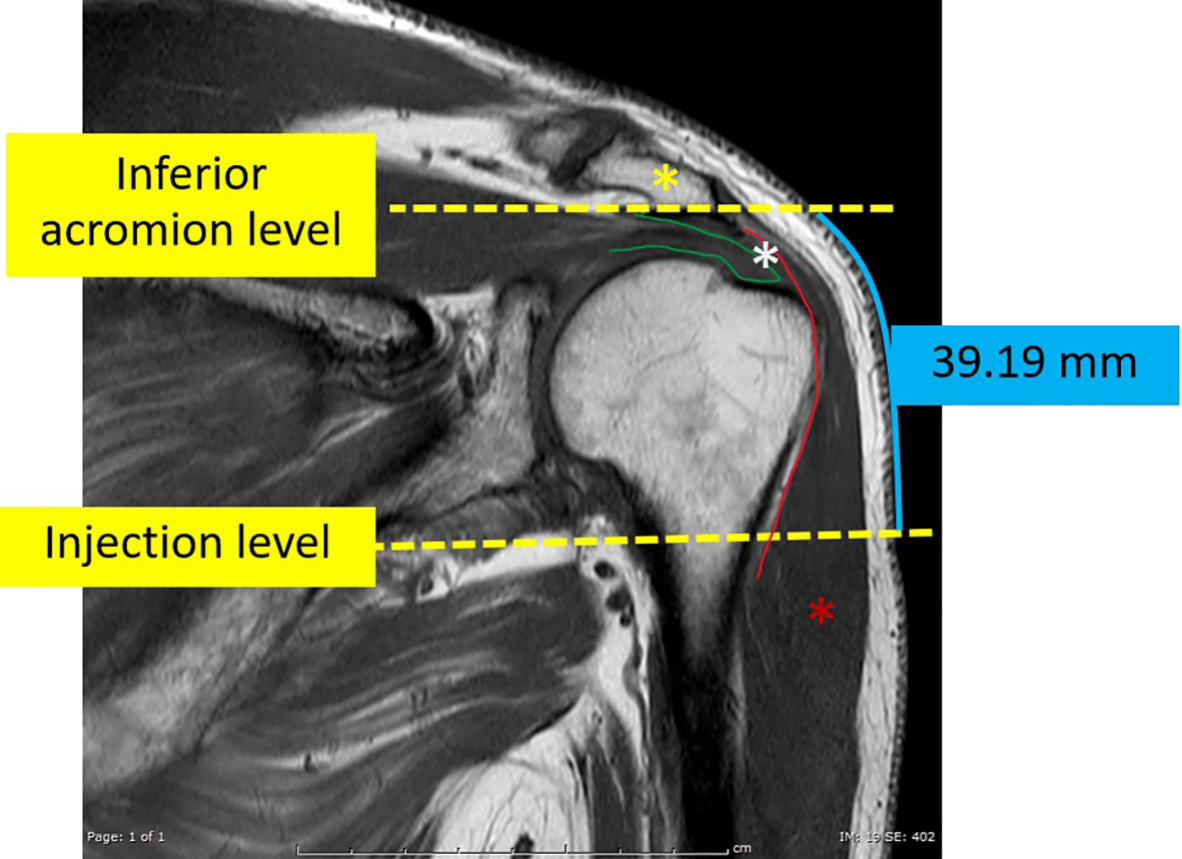
The coronal T1-weighted image of an MRI shoulder passing through the mid-acromial process (yellow asterisk) which was used as the reference coronal image. On this image, a vertical line, about 39.19 mm in length, was manually drawn vertically downwards from the level of the inferior aspect of the acromion process. The terminal point of this vertical line indicated the level of the simulated injection. The green line indicates the supraspinatus tendon. The red asterisk indicates the deltoid muscle. The red line indicates the subdeltoid fascia. The space between the deltoid and the acromial process and the underlying supraspinatus is the subacromial-subdeltoid bursa (white asterisk).

**Figure 4:**
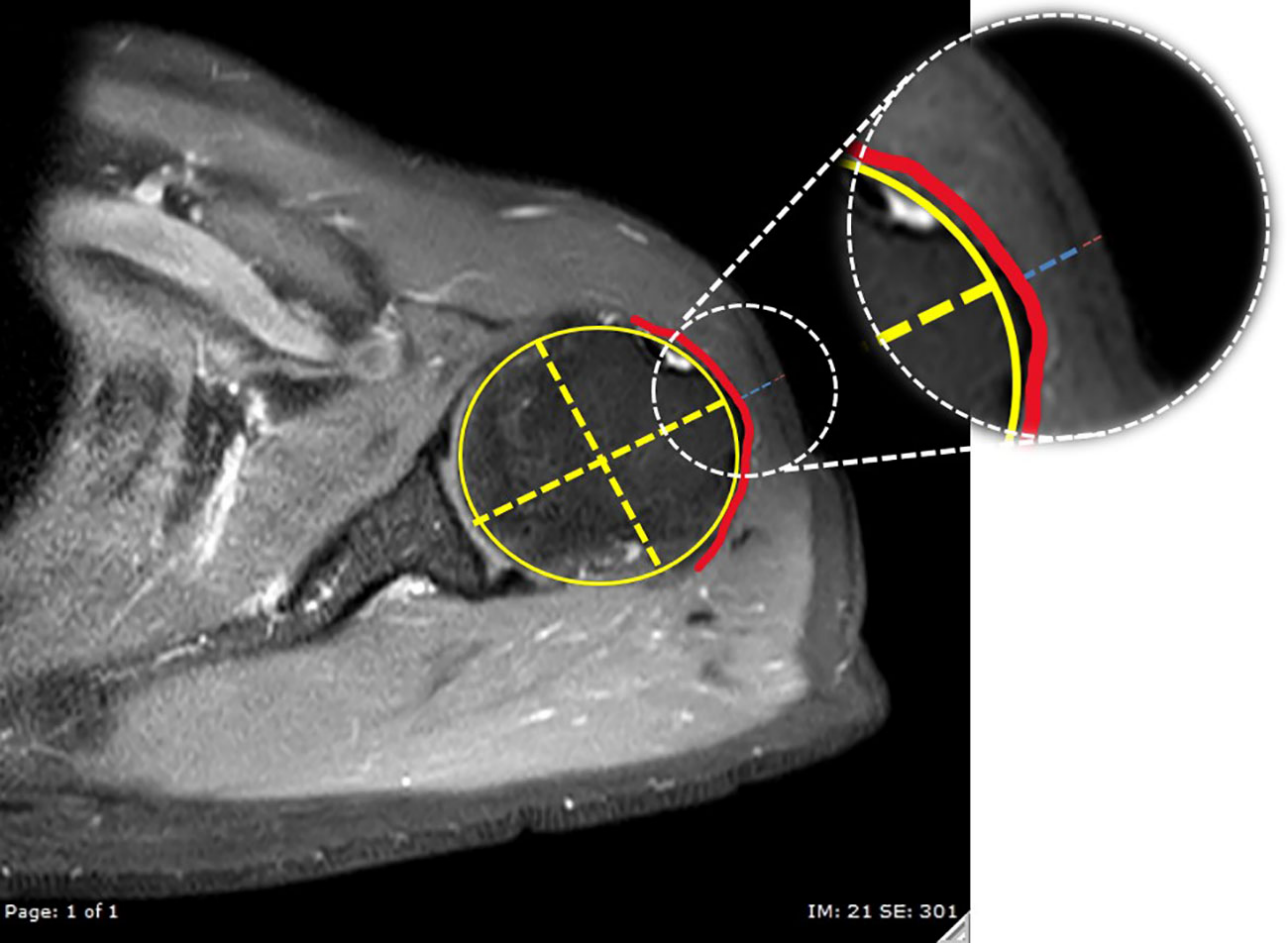
The reference axial proton density-weighted image with fat suppression at the injection level. A best-fit circle of the humeral head was created (yellow circle), then a line passing through the center of the humeral head (decussation of the yellow dashed lines) and perpendicular to the glenoid surface was used to indicate the direction for measurements of the thickness of the deltoid muscle (blue dashed line) and subcutaneous fat pad (red dashed line). The red line indicates the subdeltoid fascia.

For this investigation, MRI images that passed through the most anterolateral aspect of the acromion process, as depicted in **Figure 3**, were chosen as the coronal images. To identify the indicated site for an injection, a line of 2 fingerbreadths (39.19 mm) in length was drawn vertically downward along the skin surface, just below the inferior aspect of the acromion process. Axial proton density-weighted images at the level of the injection site were utilized for the measurements carried out in this study. The thickness of the subcutaneous fat pad and deltoid muscle measurements were obtained at designated distances from the center of the humeral head, as depicted in **Figure 4**. Each measurement was independently performed three times by an experienced musculoskeletal radiologist to ensure the accuracy and reliability of the findings.

The study’s findings are presented through the use of descriptive statistics, specifically means ± standard deviations (SDs), and incidence ratios, which indicate the penetration into the intradeltoid muscle at different needle penetration depths. Statistical analysis was conducted utilizing the R program and the epicalc package (version 3.4.3, R Foundation for Statistical Computing, Austria). To compare the mean age, mean height, mean weight, mean body mass index (BMI), mean deltoid subcutaneous fat, and mean deltoid thickness from skin to subdeltoid fascia, t-tests were employed. The chi-square test of independence or Wilcoxon rank sum test was utilized to compare the incidence ratios of penetration into the intradeltoid muscle across various needle penetration depths. Pearson’s correlation coefficient was used to assess correlations between the thickness of the deltoid subcutaneous fat pad, deltoid thickness from skin to subdeltoid fascia, deltoid muscle, and the participant’s weight, height, and BMI for both men and women. Statistical significance was set at P < 0.05. Intraclass correlation coefficients were calculated to assess measurement reliability.

## Results

509 MRI shoulder images of 222 males (123 right and 99 left shoulders) and 287 females (142 right and 145 left shoulders) were included in the study. The patient characteristics are shown in **Table 1**. Of the 509 MRI shoulders, 215 were diagnosed as rotator cuff pathology, 197 as combined rotator cuff pathology and adhesive capsulitis, 30 as osteoarthritis, 27 as adhesive capsulitis, 8 as labral pathology, 14 as combined rotator cuff and labral pathologies, 10 as combined rotator cuff and labral pathologies and adhesive capsulitis, and 8 as normal.

**Table 1:** Patient clinical and demographic characteristics.

Variable	Male (222)	Female (287)	P-value
Mean age in years (SD)	64 (11.1)	65.4 (9.4)	0.135
Mean height in cm (SD)	166.4 (6.2)	154.1 (6.6)	<0.001
Mean weight in kg (SD)	68.3 (10.1)	58 (9.2)	< 0.001
Mean BMI in kg/m^2^ (SD)	24.6 (3.3)	24.5 (3.7)	0.64
Mean thickness of subcutaneous fat pad in mm (SD)	5.4 (1.7)	8 (2.2)	< 0.001
Mean thickness from skin to subdeltoid fascia in mm (SD)	17.8 (3.4)	19.4 (3.6)	< 0.001

### Demographic data and needle lengths

**Figure 5:**
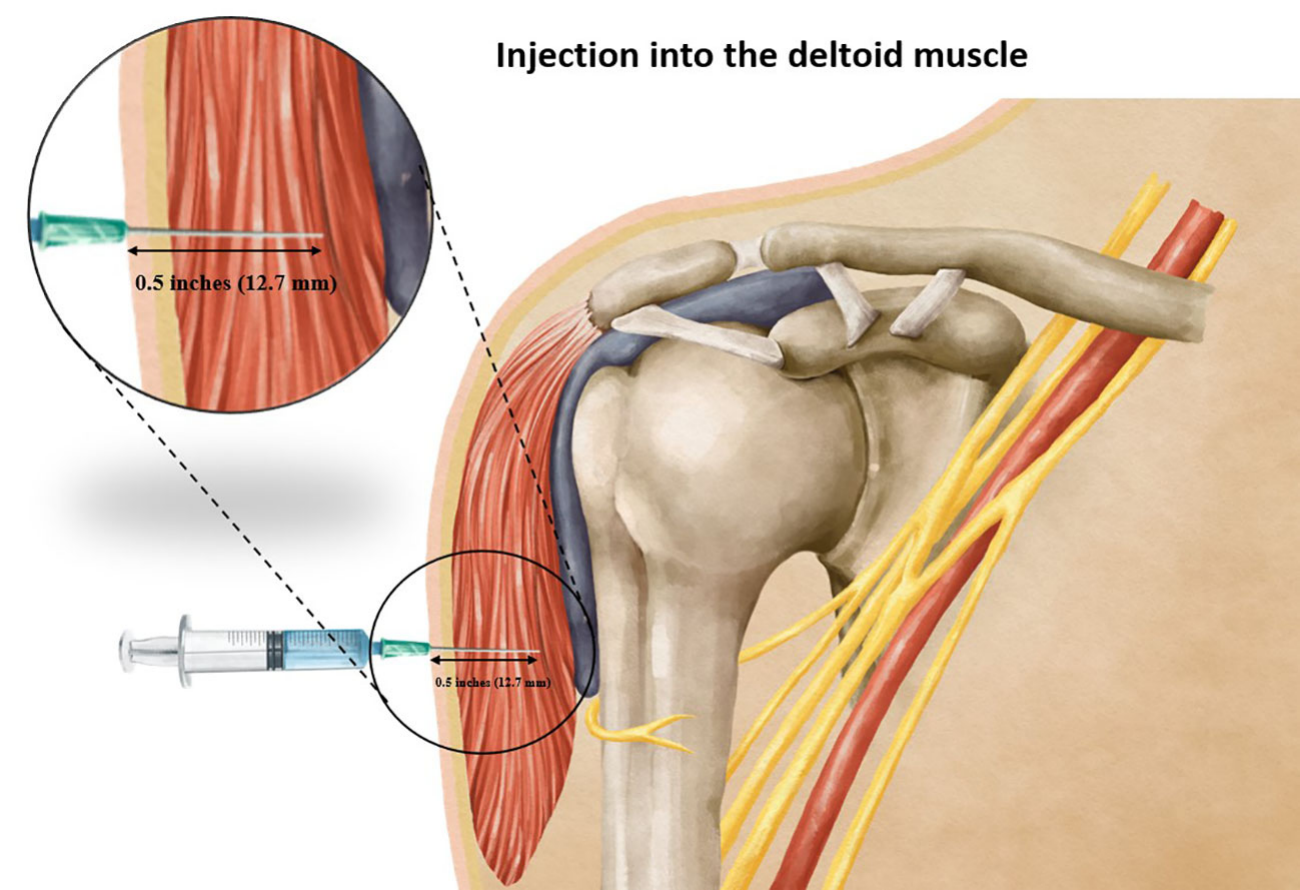
Showing the correct injection depth into the deltoid muscle when using an appropriate needle length.

The clinical and demographic characteristics of the patients are shown in **Table 1**. Our study’s findings demonstrated the outcomes of simulated vaccine administrations using different needle lengths. Notably, when using a 0.5-inch needle (12.7 mm), 100% of the simulated administrations successfully penetrated into the deltoid muscle without reaching the subdeltoid fascia (**Figure 5**). With a 1-inch needle (25.4 mm), 6 of 222 males and 17 of 287 females had needle penetration restricted to the deltoid muscle, while 216 of 222 males and 270 out of 287 females had penetration beyond the subdeltoid fascia. Employing a longer 1.5-inch needle (38.1 mm), all male and female participants had penetration beyond the subdeltoid fascia. The complete results of the spatial analysis of needle tip locations, conducted in accordance with the CDC guidelines, are displayed in **Table 2**.

**Table 2:** Final needle tip position of different needle lengths in males and females.

Depth of needle penetration	Location of needle tip																	
	Males (n=222)									Females (n=287)								
	Weight less than 70 kg (147)			Weight between 70-118 kg (75)			Weight more than 118 kg (0)			Weight less than 70 kg (263)			Weight between 70-90 kg (22)			Weight more than 90 kg (2)		
	Subcutaneous fat pad	Deltoid muscle	Subdeltoid bursa	Subcutaneous fat pad	Deltoid muscle	Subdeltoid bursa	Subcutaneous fat pad	Deltoid muscle	Subdeltoid bursa	Subcutaneous fat pad	Deltoid muscle	Subdeltoid bursa	Subcutaneous fat pad	Deltoid muscle	Subdeltoid bursa	Subcutaneous fat pad	Deltoid muscle	Subdeltoid bursa
12.7 mm (0.5 inches)	0 (0%)	147 (100%)	0 (0%)	0 (0%)	75 (100%)	0 (0%)	–	–	–	0 (0%)	263 (100%)	0 (0%)	0 (0%)	22 (100%)	0 (0%)	0 (0%)	2 (100%)	0 (0%)
25.4 mm (1 inch)	0 (0%)	0 (0%)	147 (100%)	0 (0%)	6 (8%)	69 (92%)	–	–	–	0 (0%)	13 (4.94%)	250 (95.06%)	0 (0%)	4 (18.18%)	18 (81.82%)	0 (0%)	0 (0%)	2 (100%)
38.1 mm (1.5 inches)	0 (0%)	0 (0%)	147 (100%)	0 (0%)	0 (0%)	75 (100%)	–	–	–	0 (0%)	0 (0%)	263 (100%)	0 (0%)	0 (0%)	22 (100%)	0 (0%)	0 (0%)	2 (100%)

### Correlation of the thicknesses of the skin, subcutaneous fat pad and deltoid muscle with BMI

**Figure 6:**
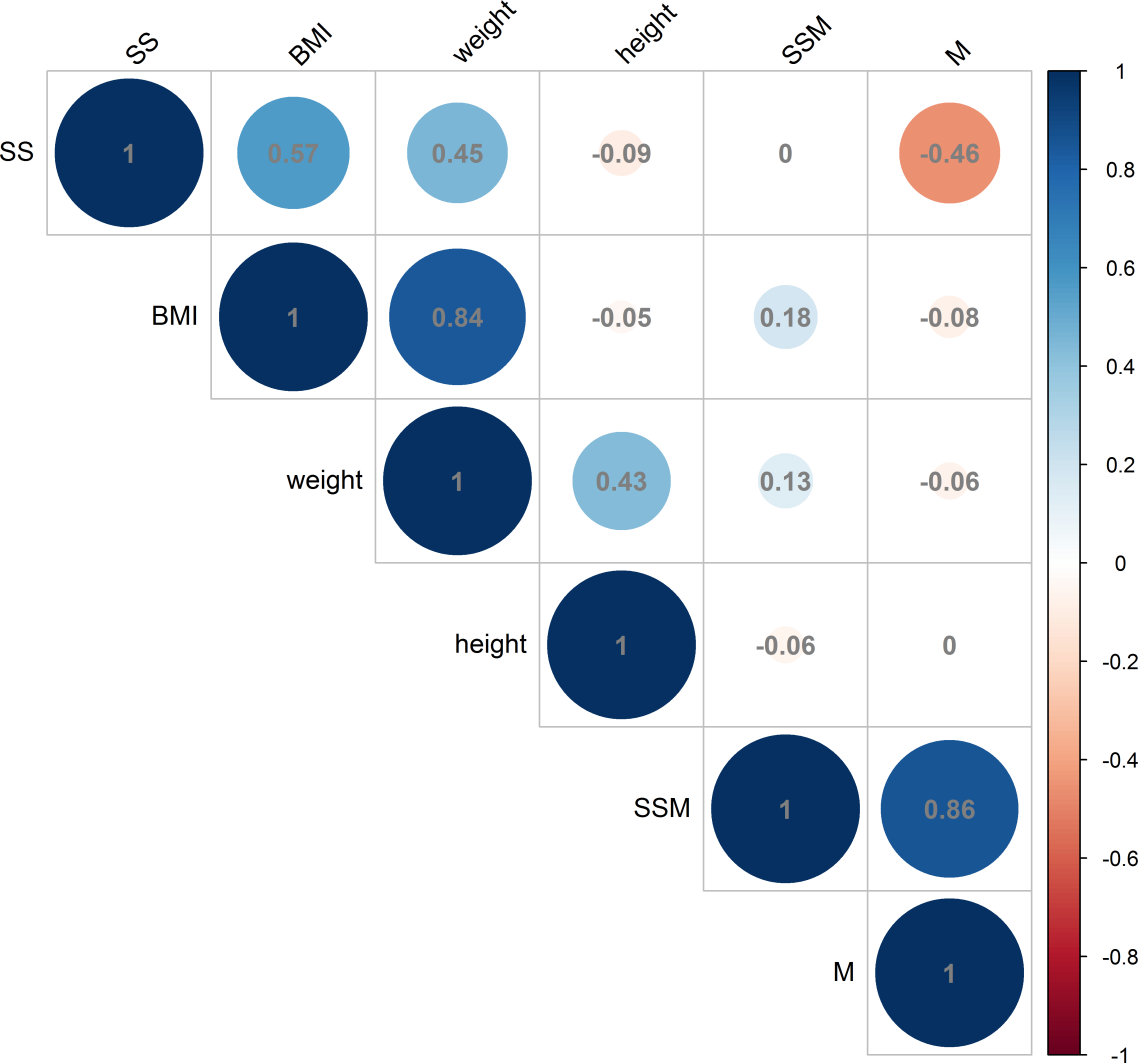
Correlations in male measurements between BMI (kg/m^2^), weight (kg) and height (cm) and the thickness of the deltoid subcutaneous fat pad, deltoid muscle and skin to subdeltoid fascia (mm). (BMI, body mass index; SS, thickness of deltoid subcutaneous fat pad; M, thickness of deltoid muscle; SSM, thickness of skin to subdeltoid fascia).

**Figure 7:**
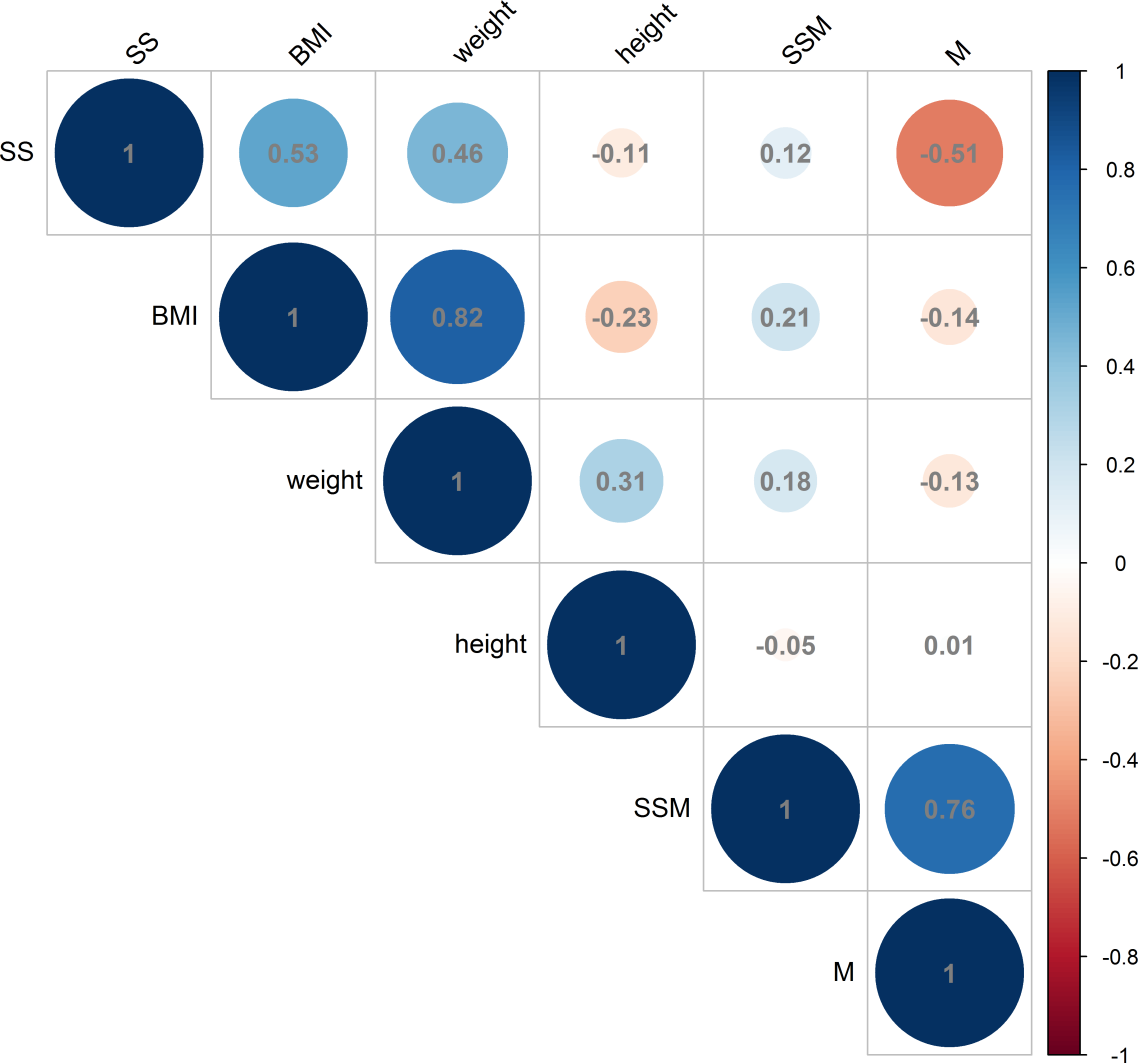
Correlations in female measurements between BMI (kg/m^2^), weight (kg) and height (cm) and the thickness of the deltoid subcutaneous fat pad, deltoid muscle and skin to subdeltoid fascia (mm). (BMI, body mass index; SS, thickness of deltoid subcutaneous fat pad; M, thickness of deltoid muscle; SSM, thickness of skin to subdeltoid fascia).

The analysis revealed a positive correlation between BMI and the thickness of the deltoid subcutaneous fat pad, and the total thickness from the skin to the subdeltoid fascia. Conversely, a negative correlation was observed between BMI and deltoid muscle thickness, as illustrated in **Figures 6**, **7**. There was high intraobserver correlation with the correlation coefficients of all assessments greater than 0.986.

## Discussion

This investigation was aimed at determining the most appropriate needle penetration depth for intradeltoid vaccination administration, seeking to achieve precise vaccine delivery within the deltoid muscle while mitigating the potential for overpenetration or injury to adjacent anatomical structures. Our study found that employing a 0.5-inch needle with a perpendicular insertion angle relative to the skin, guided by the reference point of two fingerbreadths below the mid-acromion process, yielded a 100% success rate in ensuring a vaccine injection accurately targeted the deltoid muscle (**Figure 5**). These observations hold significant implications for optimizing vaccination procedures and enhancing the safety and efficacy of intradeltoid administrations. Furthermore, by employing meticulous measurements and adherence to the recommended injection technique, healthcare practitioners can confidently administer vaccinations with enhanced precision and reduced risk of adverse events, thereby reinforcing the role of intradeltoid injections in contemporary vaccination practices.

According to the guidelines provided by the CDC on vaccine administration, the recommended injection site and needle length for intradeltoid vaccinations are as follows: The injection site should be 2-3 fingerbreadths below the mid-acromion process, aligned perpendicularly to the skin. However, in clinical practice, many medical professionals often follow a general rule of thumb and insert the needle 1-3 fingerbreadths below the acromion process. Unfortunately, this approach can lead to complications. Earlier reports have found that inserting the needle only 1 fingerbreadth below the acromion process resulted in cases of subacromial bursitis following the vaccination (4, 11). A case series of shoulder injuries related to vaccine administration (SIRVA) after a COVID-19 vaccination demonstrated the potential clinical consequences of administering vaccines at a too-high injection site. Additionally, the series highlighted the challenge of differentiating these patients from those with pre-existing but undiagnosed septic arthritis of the shoulder (4). In one instance, a vaccinated patient received a combined therapy of intravenous antibiotics and an oral non-steroidal anti-inflammatory drug (NSAID) as an empirical treatment because septic arthritis could not be ruled out during the initial evaluation. Although the patient eventually experienced complete resolution of the bursitis, the unnecessary antibiotic exposure raised concerns about the development of antibiotic resistance. Another case reported by Mayer et al. (12) involved subacromial bursitis as a complication following a COVID-19 vaccine administration at a too-high location. The patient experienced acute severe pain, disrupted daily activities, and low-grade fever. By considering the patient’s vaccination history, observing the needle injection mark indicating improper needle placement, and analyzing the clinical symptoms, the physician could diagnose SIRVA-related subacromial bursitis and offer available treatment options. Due to the patient’s desire to return to work early, arthroscopic decompression was chosen. During the procedure, a large hemorrhagic bursitis of the subacromial bursa was visualized and decompressed using an arthroscopic shaver. In another case report, a more complicated subacromial bursitis resulted from a too-high injection site of a human papilloma (HPV) vaccine (13). The strong adjuvant in the HPV vaccine caused the bursitis to be unresponsive to corticosteroid injections, leading to chronic severe inflammation evident during arthroscopy. Consequently, the patient underwent an arthroscopic synovectomy with subacromial decompression as definitive treatment.

Caution must also be exercised at 3 fingerbreadths, or approximately 2 inches (5.08 cm), below the acromion process since the axillary nerve has been identified to be at risk of puncture injury with injections made at this location. For instance, Choi et al. (14) documented a case in which an intramuscular injection of a non-neurotoxic agent, hydroxyzine hydrochloride, led to an isolated paralysis of the middle head of the deltoid. Notably, the absence of pain or sensory deficit after the injection suggested a direct injury to the motor branch of the axillary nerve rather than an injectate-related chemical neuropathy, which would likely have resulted in a more widespread extent of injury. Similarly, another case report (15) highlighted the potential injury of the axillary nerve due to too-low needle insertions. Following injections of influenza, diphtheria, and tetanus vaccines, the patient experienced limited active ranges of shoulder motion in abduction and forward flexion, along with decreased muscle power in the corresponding muscle groups. Examination of voluntary forward flexion and abduction revealed an absence of contractility in the anterior and medial heads of the deltoid, both through observation and palpation. The affected dermatome also exhibited decreased sensation, corroborating the presence of an axillary nerve lesion. The diagnosis was confirmed through axonal regeneration patterns observed on nerve conduction studies (NCS). The preferred treatment approach involved physical rehabilitation, which resulted in complete clinical resolution at 11 months post-injury, with normal NCS patterns observed at 12 months.

Given these findings, administering vaccines in this region requires careful attention to minimize the risk of nerve injury and its associated potential complications. The precise selection of needle length is important in vaccination procedures. In accordance with the CDC guidelines, specific needle lengths are recommended based on an individual’s weight. However, in our investigation concerning the application of these recommendations to our particular population and have identified various concerns regarding injection depth. Our simulation study, conducted based on the CDC guidelines for vaccine administration, yielded diverse outcomes based on different needle lengths among individuals with varying body weights. In subjects weighing less than 70 kilograms, employing a 1-inch needle resulted in complete penetration into the deltoid muscle in 0% of males and 4.94% of females. However, when simulating the use of a 1.5-inch needle in males weighing between 70 and 118 kilograms and females weighing between 70 and 90 kilograms, a 100% risk of injection into the subdeltoid bursa was observed. Moreover, in females weighing over 90 kilograms, the utilization of a 1.5-inch needle also led to a 100% risk of injection into the subdeltoid bursa. These findings unequivocally demonstrate that intradeltoid vaccinations adhering to the CDC guidelines carry a potential risk of subdeltoid bursitis attributable to the utilization of too-long needle lengths. It is thus necessary for healthcare practitioners to be mindful of individual body weights and employ appropriate needle lengths to minimize the risk of such complications.

While two fingerbreadths below the acromion process is the safest injection site to avoid axillary nerve injury, caution must also be exercised to prevent overpenetration and subdeltoid bursitis. Previous recommendations for full needle insertions in intramuscular vaccinations could lead to overpenetration in our study population. Conversely, a minimum 5-mm (1/5th inch) insertion depth, commonly recommended for intramuscular injections, may not be applicable to the Thai population, as it could result in vaccine delivery into the subcutaneous fat pad, leading to potential vaccination failure or local reactions. In terms of local reactions, various studies have reported higher complication rates in individuals who received a subcutaneous vaccination as opposed to those getting an intramuscular injection (1, 3, 16, 17). This should raise awareness of the importance of following the correct intramuscular injection technique as choosing an insufficient needle length will result in an increased possibility of a subcutaneous injection.

To address individual variability and optimize vaccination success, various measures for assessing the skin-to-deltoid muscle distance have been proposed, including age, body weight (18), and BMI (19, 20). In an ultrasound study of 386 patients, BMI was found to have a superior predictive value of the deltoid subcutaneous fat pad (DSFP) thickness (20). The study concluded that the appropriate needle depth was DSFP thickness + 7 mm, but which has the problem of being difficult to apply in a real-time vaccination setting. The previously mentioned study from Cook et al. (2006) (19) recommended a 25-mm needle for intramuscular injections in individuals with a BMI less than 35 kg/m_2_ and a 32-mm needle for females with a BMI over 35 kg/m_2_. These and other studies have various recommendations concerning appropriate needle length and injection site, which are often, however, somewhat contradictory, which may be attributed to differences in study populations, methodologies, or anatomical considerations. Clearly, optimal needle length for intramuscular injections can depend on multiple factors, including muscle mass, age, gender, and anatomical variations. Therefore, while our study suggests a shorter needle length (0.5 inches of needle penetration depth), it must be considered within the broader context of existing guidelines and individualized patient care, taking into account variations in anatomy and clinical practice, to ensure safe and effective intramuscular injections in other populations..

Our study found positive correlations between BMI and the thickness of the deltoid subcutaneous fat pad and the thickness from skin to subdeltoid fascia, but a negative correlation between BMI and deltoid muscle thickness in both males and females. Our findings are similar to the findings of earlier studies, which found that body weight relative to height was positively associated with subcutaneous fat accumulation. Earlier studies have found that as BMI increased, there was a corresponding increase in subcutaneous fat content (21, 22). Conversely, a negative correlation has been found between BMI and the thickness of the deltoid muscle, indicating that as BMI rises, the proportion of lean muscle mass, including the deltoid muscle, tends to decrease. The results of our study suggest that individuals of both male and female genders may be at an increased risk of having injections administered into the deltoid subcutaneous fat pad when shorter needle lengths are employed. Conversely, the utilization of longer needle lengths may elevate the risk of injections into the subdeltoid bursa.

For clinical implementation, we recommend the utilization of a 0.5-inch needle, which should be inserted perpendicular to the skin at an injection site of 2 fingerbreadths below the mid-acromion process. Alternatively, a 1-inch needle can be employed, with the insertion depth limited to half its length. However, caution should be exercised when using the 1-inch needle, as accurately determining the midpoint for insertion is subjective and susceptible to error. By employing this technique, it can be ensured that the needle tip is correctly inserted within the deltoid muscle, leading to a heightened success rate of vaccinations with minimal complications. These recommendations are supported by both empirical evidence and extensive research in the field of vaccination techniques. As such, their incorporation into clinical practice is likely to yield superior outcomes and contribute to improved patient care and safety. However, doctors or practitioner nurses should be cautious concerning this recommendation in extremely thin or obese patients who may have body measurements outside the normal range. Another option to avoid intradeltoid vaccinations is to use a microneedle vaccination. Microneedles for vaccination offer a promising alternative to intradeltoid vaccination, potentially mitigating complications associated with inappropriate needle penetration depth. Unlike traditional intradeltoid injections, microneedles are designed to painlessly and consistently deliver vaccines into the epidermal and dermal layers of the skin. This approach capitalizes on the abundance of antigen-presenting cells and the dense network of capillaries within the skin, leading to efficient antigen uptake and activation of the immune system. By bypassing muscle tissue, microneedles eliminate the risk of suboptimal vaccine delivery into the deltoid muscle and associated complications such as pain or discomfort, and reduced efficacy. Furthermore, microneedles may offer a more controlled and precise vaccination process, minimizing variations in injection techniques, thereby reducing the likelihood of complications (23, 24). This innovative vaccination method has the potential to improve vaccination outcomes and patient experiences while also enhancing vaccine coverage and accessibility.

This study had several strengths, particularly in considering the practical applicability of the results to real-world vaccination scenarios. To ensure accurate measurements of deltoid muscle thickness, the researchers controlled the shoulder position during the MRI scans. Specifically, they opted for a relaxed adduction position with the patient’s arm by their side, as this position was deemed more suitable compared to abduction, another common shoulder position used during vaccine administration. Abduction can lead to varying degrees of deltoid muscle contraction, making it challenging to standardize the procedure across different individuals. In mass vaccination sites, precise control of shoulder abduction degree becomes impractical, increasing the risk of errors and complications associated with improper needle penetration depths. Moreover, the researchers chose MRI over ultrasonography as the imaging modality. The MRIs provided detailed and high-resolution images of the soft tissue layers, allowing for accurate assessment of deltoid muscle thickness. Conversely, ultrasonography can result in compression effects due to the use of the ultrasound probe, potentially leading to misinterpretation of the thicknesses of the skin and subcutaneous layers. By employing these methodological considerations, the study aimed to enhance the relevance and practicality of its findings for real-world vaccination practices.

This study also had some limitations. Firstly, our findings were derived from a specific Thai population, and the variations in body fat distribution and deltoid muscle thickness observed in this study may not be applicable to populations outside of Southeast Asia. Consequently, caution should be exercised when extrapolating these results to other ethnicities and/or geographic regions. Secondly, our research design excluded individuals with extremely low or high BMI values. Therefore, the determination of appropriate needle lengths for these specific subpopulations cannot be ascertained with precision from the present study. Further investigations, specifically targeting individuals with extreme BMI values, are necessary to establish accurate recommendations for needle length in such cases. Such comprehensive investigations will provide a more nuanced understanding of vaccination techniques and contribute to enhanced clinical guidelines across a broader spectrum of individuals. Nonetheless, the present study’s insights remain valuable within the context of the examined Thai population, facilitating informed decision-making for healthcare practitioners working with this specific demographic.

## Conclusions

Our study demonstrates that using a 0.5-inch needle penetration depth perpendicular to the skin, at two fingerbreadths below the mid-acromion process is a safe and effective for intradeltoid vaccination. This technique ensures precise vaccine delivery into the deltoid muscle while minimizing the risk of overpenetration or injury to surrounding structures.

Adherence to the CDC guidelines for vaccination may lead to the use of a needle length that is too long, potentially resulting in injection into the subdeltoid bursa. Our findings highlight the importance of carefully selecting an appropriate needle length to avoid complications and ensure optimal vaccination outcomes.

## Data availability statement

The original contributions presented in the study are included in the article/**Supplementary Material**. Further inquiries can be directed to the corresponding author.

## Ethics statement

The study, involving humans, was approved by the Ethics Committee of the Faculty of Medicine of Prince of Songkla University. The study was conducted in accordance with the local legislation and institutional requirements. The Ethics Committee waived the requirement of written informed consent for participation from the participants or the participants’ legal guardians/next of kin because this was a retrospective study.

## Author contributions

TL: Conceptualization, Investigation, Methodology, Validation, Writing – review & editing. PB: Investigation, Methodology, Validation, Writing – review & editing. KP: Investigation, Methodology, Writing – review & editing. TN: Investigation, Methodology, Writing – review & editing. PK: Conceptualization, Supervision, Writing – review & editing. CC: Conceptualization, Formal Analysis, Methodology, Project administration, Writing – original draft, Writing – review & editing.
